# Pharmacological manipulations of judgement bias: A systematic review and meta-analysis

**DOI:** 10.1016/j.neubiorev.2019.11.008

**Published:** 2020-01

**Authors:** Vikki Neville, Shinichi Nakagawa, Josefina Zidar, Elizabeth S. Paul, Malgorzata Lagisz, Melissa Bateson, Hanne Løvlie, Michael Mendl

**Affiliations:** a Centre for Behavioural Biology, Bristol Veterinary School, University of Bristol, Langford BS40 5DU, United Kingdom; b Evolution and Ecology Research Centre, School of Biological, Earth and Environmental Sciences, University of New South Wales, Sydney, New South Wales, Sydney, NSW 2052, Australia; c The Department of Physics, Chemistry and Biology, IFM Biology, Linköping University, SE-581 83 Linköping, Sweden; d Institute of Neuroscience and Centre for Behaviour and Evolution, Newcastle University, Newcastle upon Tyne NE2 4HH, United Kingdom

**Keywords:** Affective state, Animal welfare, Judgement bias, Meta-analysis, Mood disorders, Systematic review

## Abstract

Validated measures of animal affect are crucial to research spanning numerous disciplines. Judgement bias, which assesses decision-making under ambiguity, is a promising measure of animal affect. One way of validating this measure is to administer drugs with affect-altering properties in humans to non-human animals and determine whether the predicted judgement biases are observed. We conducted a systematic review and meta-analysis using data from 20 published research articles that use this approach, from which 557 effect sizes were extracted. Pharmacological manipulations overall altered judgement bias at the probe cues as predicted. However, there were several moderating factors including the neurobiological target of the drug, whether the drug induced a relatively positive or negative affective state in humans, dosage, and the presented cue. This may partially reflect interference from adverse effects of the drug which should be considered when interpreting results. Thus, the overall pattern of change in animal judgement bias appears to reflect the affect-altering properties of drugs in humans, and hence may be a valuable measure of animal affective valence.

## Highlights

• Pharmacological manipulations of affective state alter judgement bias.
• The effect of the manipulations was greatest at the probe cues and negative reference cue. Multiple probe cues are recommended for future judgement bias studies.
• Anxiogenics and depressants have greater effects than antidepressants and anxiolytics.
• Antidepressant drugs targeting the adrenergic system induce a negative judgement bias.

## 1 Introduction

Measurement of affective state, which is defined as comprising both short-term emotions and longer-term moods (Mendl et al., 2010), and according to dimensional models of affect, valence and arousal components (Mendl et al., 2010), is important to a number of disciplines including psychopharmacology, neuroscience, and animal welfare science, as well as being of societal interest. For example, mood disorders are a significant global concern; it is estimated that 780,000 people died by suicide in 2015, with on average one death every 40 s (World Health Organization, 2017). Major depressive disorder is ranked as the largest single contributor to global disability, and anxiety disorders are ranked sixth (World Health Organization, 2017). The development of pharmacological treatments for mood disorders has been largely dependent on empirical studies using non-human animals (Valvassori et al., 2013, Rupniak, 2003). Reliable and validated measures of affective state in non-human animals are therefore crucial to understanding the neurobiological aetiology of these disorders and to assist in the development of novel treatments. In particular, measures should have both predictive validity (i.e. the extent to which the measure is altered in the predicted direction by drugs which alter human affect) and construct validity (i.e. the extent to which they measure precisely what they claim to measure) (Nestler and Hyman, 2010). Predictive validity is typically regarded as the ‘gold-standard’ for validating novel behavioural measures of affective state (De Vry and Schreiber, 1997, McArthur and Borsini, 2006).

Numerous behavioural assays have been developed to assess animal affect. The most common of these include the forced swim test, and its derivative the tail suspension test, which are considered to measure helplessness (Kato et al., 2007, Porsolt et al., 1977, Cryan et al., 2002, Steru et al., 1985); the sucrose preference test which is considered to measure hedonic capacity (Willner et al., 1987, Willner, 1997); and the elevated plus maze which is considered to assess the relative value of exploration to safety (Montgomery, 1955, Pellow et al., 1985). Overall, there is good evidence to suggest that these assays have predictive validity with a broad range of antidepressant or anxiolytic drugs resulting in changes in the predicted direction (i.e. increased latency to immobility; preference for greater sucrose levels; and a greater proportion of time spent in open compared to closed arms of the plus maze). However, when dosed with antidepressant drugs that are used to treat generalised anxiety disorder in humans, rodents do not consistently increase their proportion of time spent in the open compared with closed arms of the open plus maze and hence do not appear to reduce anxiety-like behaviour (Rodgers et al., 1997, Borsini et al., 2002, Carobrez and Bertoglio, 2005). Additionally, the construct validity of these assays has been disputed. For example, it has been argued that the forced swim test and tail suspension test reflect a learnt response rather than helplessness (Molendijk and de Kloet, 2015, Borsini and Meli, 1988, West, 1990, Bogdanova et al., 2013, Naudon and Jay, 2005). Similarly, research has shown that humans with depression show no reduction in their preference for sucrose over water (Dichter et al., 2010, Berlin et al., 1998) and that body weight may be a strong confounding factor in the sucrose consumption test (Forbes et al., 1996). The outlined deficiencies in currently used assays means that there is a clear need for improved methods to measure affective state in non-human animals that have both construct and predictive validity.

The judgement bias task (sometimes referred to as the cognitive bias task or ambiguous cue interpretation task) provides an alternative means to examine affect in non-human animals and has been used widely in the field of animal welfare science since its conception by Harding et al. (2004), (Bethell, 2015, Baciadonna and McElligott, 2015). The judgement bias task examines decision-making under ambiguity. Although there is some variation in methodology, the basic principles of the task outlined here are applicable to all judgement bias studies. Individuals are first trained to associate the presentation of one reference cue (e.g. a high frequency tone) with a reward and presentation of another reference cue (e.g. a lower frequency tone) with a lower reward or punisher. Once training is complete, individuals are presented with one or a few untrained probe cues that are intermediate between the reference cues (e.g. medium frequency tones). Their responses to these ambiguous cues are measured to see whether they treat them as signalling the more or less positive outcome. This is measured as latency to approach the cue or choice to execute or not execute the riskier action which could lead to either the more or less positive outcome (i.e. not the safe action which leads to the null outcome). A decreased latency to approach the cue, or more frequent execution of the riskier action (often deemed ‘more optimistic’ or ‘less pessimistic’ in the judgement bias literature), is interpreted to reflect a relatively more positive affective state.

The task is based on the empirical finding that humans experiencing anxiety and depression have a greater expectation of punishing events or reduced expectation of rewarding events than clinically healthy humans (Nygren et al., 1996, Johnson and Tversky, 1983, Wright and Bower, 1992). To assess the extent to which judgement bias could measure subjective affective state in humans, the task has been back-translated to human subjects. Studies using the back-translated judgement bias task have demonstrated a correlation between judgement bias and measures of subjectively-experienced affect, such as the State-Trait Anxiety Inventory (STAI), Visual Analogue Scale for Anxiety (VAS-A), and negative affect dimension of the Positive and Negative Affect Schedule (PANAS) (Paul et al., 2011, Anderson et al., 2012, Iigaya et al., 2016). The finding that judgement bias correlates with subjective reports of affective state in humans supports judgement bias as measure of affect, and hence the task appears to have strong construct validity.

A well-established and widely-used approach to validating behavioural measures of affect in non-human animals, which will be used here to assess the validity of judgement bias as a potential measure of affect, is to assess whether drugs with known affect-altering properties in humans produce the predicted shift in the behavioural measure when administered to non-human animals (Kara et al., 2017, Yankelevitch-Yahav et al., 2015, Cryan et al., 2005, Willner, 1984). Specifically, we ask whether pharmacological induction of neurobiological states associated with relatively positive or negative reported affect in humans produces the predicted effect on judgement bias in animals. It is thus important to consider the mechanisms by which such pharmacological manipulations might alter behaviour. There are several cognitive mechanisms that have been proposed to underlie judgement biases and these include changes in attention, perception, reward and punisher sensitivity, prior expectation of rewards and punishers, and action selection (see (Mendl et al., 2009) for review). Computational analyses of judgement bias data have suggested that both an individual's sensitivity to rewards and punishers and prior expectation of rewards and punishment are key sources of variation in judgement bias (Iigaya et al., 2016). The neurobiological systems underlying these processes and decision-making in general have been subject to much investigation (Doya, 2008, Daw and Doya, 2006, Rangel et al., 2008, Mendl et al., 2009). Briefly, dopaminergic, adrenergic, glutaminergic, and GABAergic activity (particularly in the medial prefrontal cortex, anterior cingulate cortex, thalamus, and locus coeruleus) have been widely implicated in attention and perception (Burk et al., 2018, Logue and Gould, 2014, Posner et al., 1988, García-Cabezas et al., 2007), serotoninergic, dopaminergic, opioidergic, and GABAergic activity (particularly in the orbitofrontal cortex, nucleus accumbens, mesolimbic dopamine projections, and amygdala) have been widely suggested to encode the value and probability of rewards and punishers (Owens and Nemeroff, 1994, Boureau and Dayan, 2011, Mendl et al., 2009, Rangel et al., 2008, Berridge et al., 2009), and adrenergic, serotinergic, and dopaminergic activity (particularly in the basal ganglia and locus coeruleus) have been widely implicated in action selection (Aston-Jones and Cohen, 2005, Best et al., 2013, Reiner et al., 1998). Noteably, these neurobiological systems are also considered to play a role in human mood disorders (see Box 1). Furthermore, neurotransmitter receptor systems are highly conserved across species (Ottaviani and Franceschi, 1996, Venter et al., 1988, Jorgensen, 2014) and non-pharmacological manipulations designed to induce a positive or negative affective state in non-human animals also result in changes in the activity of these systems (Ramboz et al., 1998, Bezard et al., 2003, Laviola et al., 2008, Haenisch et al., 2009). Hence, there are a number of routes by which pharmacologically-induced neurobiological states associated with relatively positive and negative affect (as outlined in Box 1) might alter judgement bias.

Box 1 **Neurobiological targets of affect-altering drugs, i.e. those with antidepressant, depressant, anxiolytic, or anxiogenic effects:**

*Adrenergic system*: Epinephrine and norepinephrine are both hormones and neurotransmitters that bind to adrenergic receptors. The adrenergic system is involved in the early stages of a stress response (Sapolsky et al., 2000). Brains of depressed patients have reduced levels of norepinephrine and antidepressant drugs such as reboxetine selectively-target the adrenergic system (Klimek et al., 1997, Massana, 1998).

*Dopaminergic system*: Dopamine is a neurotransmitter and neuromodulator that can have both inhibitory and excitatory effects on target dopamine neurons. Dopaminergic-system dysregulation is associated with depression (Papakostas, 2006). Antidepressant drugs targeting dopamine (although non-specifically) such as monoamine oxidase inhibitors (MAOIs) are available but limited in clinical usage (Papakostas, 2006).

*Gamma-Aminobutyric acid (GABA) system*: The neurotransmittor GABA, which binds to GABA receptors, is the major inhibitory neurotransmitter in the brain (Nemeroff, 2003). Reduced GABA levels are associated with panic disorder (Goddard et al., 2001, Nemeroff, 2003). A number of commercially available treatments for anxiety disorders, such as barbituates, benzodiazepines, and gabapentins, are purported to work by enhancing GABA function.

*Glucocorticoid system*: Glucocorticoids are a class of steroid hormones that bind to glucocorticoid receptors. The system is involved in the later stages of a stress response, altering cognitive functioning, such as attention and memory, following an acute stressor (Sapolsky et al., 2000). Elevated secretion of the glucocorticoid cortisol, specifically upon waking, has been proposed as a biomarker of depression (Dienes et al., 2013, Goodyer et al., 2009).

*Glutaminergic system*: Glutamate is the brain's major excitatory neurotransmitter and targets glutaminergic receptors that include NMDA, AMPA, and kainite (D’Souza, 2015). Several recreational dissociative drugs target the glutaminergic system specifically, such as ketamine and phencyclidine (PCP). NMDA receptor antagonists, such as ketamine, have been found to have antidepressant effects (Berman et al., 2000, Papp and Moryl, 1994).

*Opioid system*: Opioid receptors are targeted by a number of neuropeptides including endorphins and nociceptin. The opioid system plays a key role in pain modulation, and mediates the euphoric (mood-improving) and analgesic effects of a number of recreational and clinical drugs such as morphine and heroin (Corbett et al., 2006).

*Oxytocin system*: Oxytocin is a hormone and neuropeptide that targets the oxytocin receptor. The oxytocin system has been implicated in depression; low oxytocin levels have been observed in depressed patients (Frasch, 1995).

*Serotoninergic system*: Serotonin is a primarily inhibitory neurotransmitter that binds to serotonergic receptors (Owens and Nemeroff, 1994). There is a wealth of evidence indicating a link between low levels of serotonin and depression (Owens and Nemeroff, 1994). Antidepressant drugs that target the serotonergic system, such as citalopram and fluoxetine, are commonly prescribed (Mars et al., 2017).

Alt-text: Box 1

Research has been conducted to assess how judgement bias is influenced by affect-altering drugs in non-human animals (See Table 1). Synthesis of these studies would provide an important first step to determine the ability of the judgement bias task to measure pharmacologically-induced neurobiological states associated with positive or negative affect, and hence elucidate the potential validity and reliability of judgement bias as a measure of affect in non-human animals. To this end, we conducted a systematic review and meta-analysis to assess whether pharmacological manipulations alter judgement bias and hence assess the predictive validity of the task. In addition to assessing whether there was an overall effect, we investigated whether the relationship between affect-altering drugs and judgement bias was moderated by factors relating to the drug and administration of the drug, such as the duration and timing of administration, dosage, and neurobiological target of the drug (see Box 1). The potential moderating effects of several task-related factors, such as the presented cue, species used, sex, reinforcement type, response type, and the outcome measure, were also investigated. While we predicted that the effects of judgement bias would be greatest at the ambiguous cues and would depend on dosage, we did not predict that the other moderators would influence the effect of the pharmacological manipulations on judgement bias.

**Table 1:** Articles included at full-text screening and reason for exclusion, where relevant.

Article number	Status	Authors	Article title	Journal	Year	Reason for exclusion
1	Included	Anderson, M. H., Munafo, M. R., Robinson, E. S. J.	Investigating the psychopharmacology of cognitive affective bias in rats using an affective tone discrimination task	Psychopharmacology	2013	NA
2	Included	Destrez, A., Deiss, V., Belzung, C., Lee, C., Boissy, A.	Does reduction of fearfulness tend to reduce pessimistic-like judgment in lambs?	Applied Animal Behaviour Science	2012	NA
3	Included	Doyle, R. E., Hinch, G. N., Fisher, A. D., Boissy, A., Henshall, J. M., Lee, C.	Administration of serotonin inhibitor p-Chlorophenylalanine induces pessimistic-like judgement bias in sheep	Psychoneuroendocrinology	2011	NA
4	Included	Enkel, T., Gholizadeh, D., Von Bohlen Und Halbach, O., Sanchis-Segura, C., Hurlemann, R., Spanagel, R., Gass, P., Vollmayr, B.	Ambiguous-cue interpretation is biased under stress-and depression-like states in rats	Neuropsychopharmacology	2010	NA
5	Included	Golebiowska, G., Rygula, R.	Effects of acute dopaminergic and serotonergic manipulations in the ACI paradigm depend on the basal valence of cognitive judgement bias in rats	Behavioural Brain Research	2017	NA
6	Included	Hales, C. A., Robinson, E. S. J., Houghton, C. J.	Diffusion modelling reveals the decision making processes underlying negative judgement bias in rats	PLoS One	2016	NA
7	Included	Hales, C.A.; Houghton, C.J.; Robinson, E.S.J.	Behavioural and computational methods reveal differential effects for how delayed and rapid onset antidepressants effect decision making in rats	European Neuropsychopharmacology	2017	NA
8	Included	Hymel, K. A., Sufka, K. J.	Pharmacological reversal of cognitive bias in the chick anxiety-depression model	Neuropharmacology	2010	NA
9	Included	Iyasere, O. S., Beard, A. P., Guy, J.H, Bateson, M.	Elevated levels of the stress hormone, corticosterone, cause “pessimistic” judgment bias in broiler chickens	Scientific Reports	2017	NA
10	Included	Kis, A., Hern di, A., Kanizs r, O., G csi, M., Top l, J.	Oxytocin induces positive expectations about ambivalent stimuli (cognitive bias) in dogs	Hormones and Behavior	2015	NA
11	Included	McGuire, M. C., Williams, K. L., Welling, L. L. M., Vonk, J.	Cognitive bias in rats is not influenced by oxytocin	Frontiers in Psychology	2015	NA
12	Included	Rygula, R., Golebiowska, J., Kregiel, J., Holuj, M., Popik, P.	Acute administration of lithium, but not valproate, modulates cognitive judgment bias in rats	Psychopharmacology	2015	NA
13	Included	Rygula, R., Papciak, J., Popik, P.	The effects of acute pharmacological stimulation of the 5-HT, na and DA systems on the cognitive judgement bias of rats in the ambiguous-cue interpretation paradigm	European Neuropsychopharmacology	2014	NA
14	Included	Rygula, R., Szczech, E., Kregiel, J., Golebiowska, J., Kubik, J., Popik, P.	Cognitive judgment bias in the psychostimulant-induced model of mania in rats	Psychopharmacology	2015	NA
15	Included	Rygula, R., Szczech, E., Papciak, J., Nikiforuk, A., Popik, P.	The effects of cocaine and mazindol on the cognitive judgement bias of rats in the ambiguous-cue interpretation paradigm	Behavioural Brain Research	2014	NA
16	Included	Sahin, C., Doostdar, N., Neill, J, C.	Towards the development of improved tests for negative symptoms of schizophrenia in a validated animal model	Behavioural Brain Research	2016	NA
17	Included	Stracke J., Ottena,W., Tuchscherer A., Puppe, B., Dupjan, S.	Serotonin depletion induces pessimistic-like behavior in a cognitive bias paradigm in pigs	Physiology and Behavior	2017	NA
18	Included	Stracke, J., Otten, W., Tuchscherer, A., Witthahn, M., Metges, C.C., Puppe, B., Dupjan, S.	Dietary tryptophan supplementation and affective state in pigs	Journal of Veterinary Behaviour	2017	NA
19	Included	Verbeek, E., Ferguson, D., Lee, C.	Are hungry sheep more pessimistic? The effects of food restriction on cognitive bias and the involvement of ghrelin in its regulation	Physiology and Behavior	2014	NA
20	Included	Verbeek, E., Ferguson, D., Quinquet de Monjour, P., Lee, C.	Generating positive affective states in sheep: The influence of food rewards and opioid administration	Applied Animal Behaviour Science	2014	NA
21	Not included	Anderson, M.H., Munafo, M.R., Robinson E.S.J.	The effects of acute psychopharmacological treatments on cognitive affective bias in rats	European Neuropsychopharmacology	2012	Conference abstract that duplicates Anderson et al 2013
22	Not included	Hales C., Bartlett J., Arban R., Hengerer B., Robinson E.	Targeted infusions with rapid acting antidepressants reveal a role for the prefrontal cortex in mediating affective biases and decision making	Brain and Neuroscience Advances	2019	Data not available
23	Not included	Karagiannis, C.I., Burman, O.H.P., Mills, D.S.	Dogs with separation-related problems show a “less pessimistic” cognitive bias during treatment with fluoxetine (ReconcileTM) and a behaviour modification plan	BMC Veterinary Research	2015	Data not available
24	Not included	Kregiel J., Golebiowska J., Popik P., Rygula R.	Dopamine induces an optimism bias in rats-pharmacological proof for the translational validity of the ambiguous-cue interpretation test	Behavioural Brain Research	2016	Retracted by author
25	Not included	Kregiel, J., Malek, N., Popik, P., Starowicz, K., Rygula, R.	Anandamide mediates cognitive judgement bias in rats	Neuropharmacology	2016	Retracted by author
26	Not included	Neill J., Gaebel W., Wolwer W., Toeller V.	NMDA receptor antagonists in rodents, relevance to negative symptoms of schizophrenia: A translational link to humans	European Archives of Psychiatry and Clinical Neuroscience	2015	Not experimental research article
27	Not included	Phillips B.U., Dewan S., Nilsson S.R.O., Robbins T.W., Heath C.J., Saksida L.M., Bussey T.J., Alsio J.	Selective effects of 5-HT2C receptor modulation on performance of a novel valence-probe visual discrimination task and probabilistic reversal learning in mice	Psychopharmacology	2018	Did not use a variant of Harding et al's task
28	Not included	Sahin, C., Podda, G., Grayson, B., Marsh, S., Aricioglu, F., Neill, J.C.	The deficit in anticipatory motivation as a negative symptom of schizophrenia: Phencyclidine treated rats exhibit pessimism in an optimistic bias task	European Neuropsychopharmacology	2015	Conference abstract that duplicates Sahin et al 2016

## 2 Methods

### 2.1 Ethics statement

Although no animal experiments were conducted directly for the purpose of this meta-analysis, data originating from studies using animals were obtained and analysed. These studies all received ethical approval from the institution at which the research was conducted.

### 2.2 Literature search

**Fig. 1:**
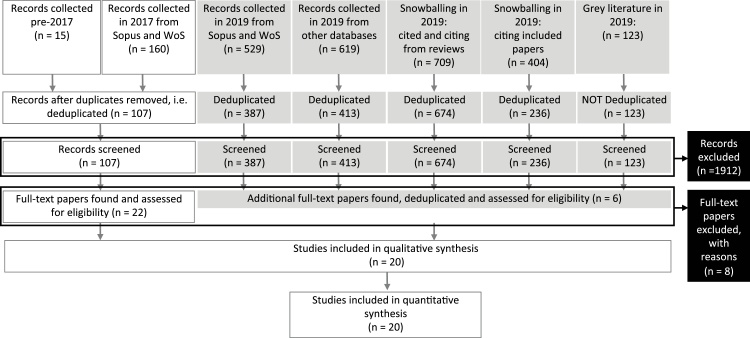
PRISMA Flow Diagram illustrating the number (*n*) of articles included at each stage of the literature review.

This study followed the Preferred Reporting Items for Systematic reviews and Meta-Analyses (PRISMA) statement (see Fig. 1) (Moher et al., 2009). A literature search was first conducted on the 2nd November 2016 to identify all judgement bias studies; the research articles from this literature search were split into groups of those that used pharmacological manipulations (to be analysed here) and those that did not (to be analysed in a separate analysis by Nakagawa et al. (*in prep.*)). These meta-analyses were conducted separately as they assessed different research questions; here we specifically want to examine the ability of judgement bias to detect pharmacological manipulations proposed to alter affect and to better understand the factors moderating this, but also due to the complexity the use of different drug doses adds the meta-analysis.

In addition to these articles, a literature search was conducted on the 13th November 2017 using Scopus and Web of Science to identify more recent research papers, and a further literature search was conducted on the 12th July 2019 using Scopus and Web of Science, as well as additional searches in other subject databases (including PsycINFO, PsycARTICLES, PsycBOOKS, PsycEXTRA, PsycTESTS, EMBASE and Medline), grey literature (using ProQuest Dissertation and Thesis Database, Google Data Search and Dimensions platform), and snowballing from reviews on the topic (cited and citing references collected) and from already included papers (citing references collected). Further details on the literature search, including the search-terms used, can be found in the supplementary material.

### 2.3 Inclusion and exclusion criteria

Following removal of duplicates, the identified articles (Fig. 1) were first screened solely by their abstract. During this abstract-based selection, articles were selected for tentative inclusion in the meta-analysis if they were deemed to be an empirical study which compared judgement bias between at least one control group and at least one treatment group to whom an affect-altering drug had been administered. Additionally, to be included, these studies had to be conducted on vertebrate non-human animals. In this analysis, an affect-altering drug was classified as any substance that was considered to have antidepressant, depressant, anxiolytic, or anxiogenic effects in humans. Where the terms affect-altering, antidepressant, depressant, anxiolytic, or anxiogenic are used throughout this article, they describe the known effect of a drug in human subjects and putative effect of the drug in non-human subjects. Twenty-eight articles met these inclusion criteria.

In the full-text screening, articles were selected on the basis that they had used a variant of Harding et al.'s (2014) cognitive judgement bias task to compare judgement bias between a group of individuals to whom a vehicle substance had been administered and at least one treatment group who had been given an affect-altering drug (Harding et al., 2004). To be included, the outcome measure had to be either latency to approach the cue (e.g. a location in a test arena) on each trial, where approaching the presented cue had been associated with reward and hence shorter latencies would be interpreted as more risk-seeking/less risk-averse behaviour (deemed ‘greater optimism’ or ‘positive judgement bias’ within the judgement bias literature), or the proportion of positive responses to each presented cue where a greater proportion would be interpreted as more risk-seeking/less risk-averse behaviour (deemed ‘greater optimism’ or ‘positive judgement bias’ within the judgement bias literature), or an outcome measure that could be converted into either form. For example, if the article reported the proportion of negative responses to each presented cue or reported the percentage of positive responses to each presented cue, the extracted data would be subtracted from one or divided by 100 respectively. All included articles either reported the proportion or latency, but not both, and hence only one of these measures was extracted for each article. Two articles were excluded at the full-text selection stage following retraction by their authors, two were excluded for not meeting the inclusion criteria, and a further two were excluded as they were conference abstracts that duplicated data presented in a journal article which was included in the analysis (Table 1). In addition to these six exclusions, two authors did not provide the requested data and so data from their articles could not be included in the meta-analysis (Table 1). A total of 20 articles were included in the meta-analysis (Table 1).

### 2.4 Data extraction

We extracted the mean and standard deviation of either the latency to approach the presented cue, or proportion of positive responses to the presented cue, as well as the sample size (number of subjects), for every pharmacological treatment and control group for each cue from each article (JZ and VN extracted the data which were checked by VN and SN). Data in a graphical format were extracted using GraphClick 3.0.3 (http://www.arizona-software.ch/graphclick/) or WebPlotDigitizer 4.1 (http://automeris.io/WebPlotDigitizer). As we extracted mean values, we acknowledge that there may have been variation in how the authors incorporated non-responses into their calculation of latency mean values which we cannot control for. If multiple drug doses had been used, these variables (i.e. mean, standard deviation, and sample size) were extracted for each dosage, and similarly, if there were test sessions that varied the duration between administration and testing or number of days of chronic drug administration, these variables were extracted for each test session. Data collected from both a vehicle and treatment group prior to drug administration were not included as these data did not provide information about the effect of the pharmacological manipulation on judgement bias.

The extracted treatment and control group data were categorised according to whether the pharmacological manipulation was expected to induce a neurobiological state associated with either a more or less positively-valenced affective state, which the judgement bias test is predicted to measure. If an anxiogenic or depressant substance had been administered, as determined by the hypotheses stated in the published article alongside the information outlined in Box 1, the treatment group was categorised as the less positive group, and the vehicle group was categorised as the more positive group (i.e. a relatively negative judgement bias was predicted in the treatment group relative to the control group). If an anxiolytic or antidepressant drug had been administered, which was also determined by the hypotheses stated in the article alongside the information outlined in Box 1, the treatment group was categorised as the more positive group and the vehicle group categorised as the less positive group (i.e. a relatively positive judgement bias was predicted in the treatment group relative to the control group). If no hypotheses were stated in the article, this categorisation was based on the description and pharmacodynamics of the substance as outlined on the DrugBank database (Wishart et al., 2017) in addition to the information presented in Box 1. Where multiple doses had been administered, higher doses of anxiolytic or anxiogenic drugs were categorised as more positive whereas higher doses of anxiogenic or depressant drugs were categorised as less positive. This was based on the widespread finding that drugs exert greater effects at higher doses (Thase, 1998, Shaheed et al., 2016).

Information about the article and authors, drug and drug administration, and methodology were also extracted (Table 1, Table 2). These included; the article title (Table 1), institute or university at which the research was conducted (extracted but not shown in Tables 1 or 2), the name of the drug (Table 2), the dosing duration (chronic – where drugs were administered repeatedly, acute – where the drug was administered immediately before testing, or chronic wash-out – the period after drug administration had stopped following chronic administration), the time between administration and testing (acute studies only), the number of days since the first dose (chronic studies only), the dosage (in mg/kg), the neurobiological target of the drug, the pharmacological manipulation type (antidepressant/anxiolytic or depressant/anxiogenic), the species tested, and the outcome variable used (latency or proportion), cue (positive reference cue, midpoint probe cue, negative reference cue, and where included the near negative probe cue and near positive probe cue) (not shown in Tables 1 or 2, although number of probe cues given instead), sex of the experimental subjects (all male, all female, or both male and female), reinforcement type used for the reference cue (reward-punishment – where the positive reference cue was rewarded and negative reference cue punished; reward-null – where the positive reference cue was rewarded and negative reference cue was not rewarded; or reward-reward – where the positive reference cue was rewarded with a high reward and negative reference cue was reward with a low reward), response type which reflected whether both or only one of the reference cues required an approach response (go/no-go – where the positive reference cue required an active response and the negative reference cue required no response, or go/go – where both reference cues required an active response), the proportion of probe trials in relation to the total number of trials, and cue type (reference or probe) (Table 2). To ensure that dosage was comparable between substances and species, each drug dose within a species was standardized by dividing the dosage (in mg/kg) by the standard deviation of all doses administered within each drug for each species.

**Table 2:** Information extracted from each article included in the analysis.

Article number	Drug	Pharmacological target	Doses	Dosing frequency	Time between administration and testing	Number of administration days prior to testing	Number of administration days between final treatment and testing	Manipulation	Species	Sex	Response type	Reinforcement type	Outcome variable	Number of probe cues	Proportion of probe cues
1	diazepam	GABAergic system	0, 0.3, 1	acute	30	NA	NA	antidepressant/anxiolytic	rat	male	go/go	reward/punisher	proportion	1	0.3
	fluoxetine	serotinergic system	0, 1	chronic	NA	1,4,8,11,15,18	NA	antidepressant/anxiolytic	rat	male	go/go	reward/punisher	proportion	1	0.3
	fluoxetine	serotinergic system	0, 1	chronic (wash-out)	NA	NA	5,7	antidepressant/anxiolytic	rat	male	go/go	reward/punisher	proportion	1	0.3
	fluoxetine	serotinergic system	0, 0.3, 1, 3	acute	60	NA	NA	antidepressant/anxiolytic	rat	male	go/go	reward/punisher	proportion	3	0.3
	reboxetine	adrenergic system	0, 0.1, 0.3, 1	acute	30	NA	NA	antidepressant/anxiolytic	rat	male	go/go	reward/punisher	proportion	3	0.3
2	diazepam	GABAergic system	0, 0.1	acute	10,180	NA	NA	antidepressant/anxiolytic	sheep	female	go/no-go	reward/punisher	latency	3	0.6
3	p-Chlorophenylalanine	serotinergic system	0, 40	chronic	NA	3,5	NA	depressant/anxiogenic	sheep	female	go/no-go	reward/punisher	proportion	3	0.6
	p-Chlorophenylalanine	serotinergic system	0, 40	chronic (wash-out)	NA	NA	5	depressant/anxiogenic	sheep	female	go/no-go	reward/punisher	proportion	3	0.6
4	corticosterone-HBC complex + reboxetine	multiple	0, 0.5 (cort) + 15 (rbx)	acute	30 (cort) + 60 (rbx)	NA	NA	depressant/anxiogenic	rat	male	go/go	reward/punisher	proportion	3	0.25
5	escitalopram	serotinergic system	0, 0.5, 1, 2	acute	30	NA	NA	antidepressant/anxiolytic	rat	male	go/go	reward/punisher	proportion	1	0.2
	haloperidol	dopaminergic system	0, 0.01, 0.02 0.05	acute	30	NA	NA	depressant/anxiogenic	rat	male	go/go	reward/punisher	proportion	1	0.2
	l-dopa	dopaminergic system	0, 2, 4, 8	acute	30	NA	NA	antidepressant/anxiolytic	rat	male	go/go	reward/punisher	proportion	1	0.2
6	FG7142	GABAergic system	0, 3.0, 5.0	acute	30	NA	NA	depressant/anxiogenic	rat	male	go/go	reward/reward	proportion	1	0.3
7	fluoxetine	serotinergic system	0, 0.3, 1	acute	30	NA	NA	antidepressant/anxiolytic	rat	male	go/go	reward/reward	proportion	1	0.3
	fluoxetine	serotinergic system	0, 1	chronic		1,4,8,11,15,18	NA	antidepressant/anxiolytic	rat	male	go/go	reward/reward	proportion	1	0.3
	fluoxetine	serotinergic system	0, 1	chronic (wash-out)	NA	4,7	NA	antidepressant/anxiolytic	rat	male	go/go	reward/reward	proportion	0.3	
	reboxetine	adrenergic system	0, 0.3, 1	acute	30	NA	NA	antidepressant/anxiolytic	rat	male	go/go	reward/reward	proportion	1	0.3
	venlafaxine	multiple	0, 1, 3	acute	30	NA	NA	antidepressant/anxiolytic	rat	male	go/go	reward/reward	proportion	1	0.3
	ketamine	multiple	0, 0.3, 1, 3	acute	60	NA	NA	antidepressant/anxiolytic	rat	male	go/go	reward/reward	proportion	1	0.3
	phencyclidine	multiple	0, 0.3, 1, 3	acute	40	NA	NA	antidepressant/anxiolytic	rat	male	go/go	reward/reward	proportion	1	0.3
	amphetamine	multiple	0, 0.1, 0.3	acute	15	NA	NA	antidepressant/anxiolytic	rat	male	go/go	reward/reward	proportion	1	0.3
	cocaine	multiple	0, 0.3, 1, 3	acute	10	NA	NA	antidepressant/anxiolytic	rat	male	go/go	reward/reward	proportion	1	0.3
8	imipramine	multiple	0, 15	acute	15	NA	NA	antidepressant/anxiolytic	chicken	male	go/no-go	reward/punisher	latency	2	0.5
	clonidine	adrenergic system	0, 0.1	acute	15	NA	NA	antidepressant/anxiolytic	chicken	male	go/no-go	reward/punisher	latency	2	0.5
9	corticosterone	glucocorticoid system	0, 4	chronic	NA	3,4,5	NA	depressant/anxiogenic	chicken	female	go/no-go	reward/punisher	latency	3	0.3
10	oxytocin	oxytocin system	NA	acute	40	NA	NA	antidepressant/anxiolytic	dog	mixed	go/no-go	reward/null	latency	1	0.3
11	oxytocin	oxytocin system	0, 0.001	acute	5	NA	NA	antidepressant/anxiolytic	rat	male	go/no-go	reward/punisher	latency	1	0.25
12	lithium chloride	multiple	0, 10, 50, 100	acute	30	NA	NA	antidepressant/anxiolytic	rat	male	go/go	reward/punisher	proportion	1	0.2
	valproic acid	GABAergic system	0, 100, 200, 400	acute	30	NA	NA	antidepressant/anxiolytic	rat	male	go/go	reward/punisher	proportion	1	0.2
13	citalopram	serotinergic System	0, 1, 5, 10	acute	30	NA	NA	antidepressant/anxiolytic	rat	male	go/go	reward/punisher	proportion	1	0.2
	d-amphetamine	multiple	0, 0.1, 0.5, 1	acute	30	NA	NA	antidepressant/anxiolytic	rat	male	go/go	reward/punisher	proportion	1	0.2
	desipramine	multiple	0, 1, 2, 5	acute	30	NA	NA	antidepressant/anxiolytic	rat	male	go/go	reward/punisher	proportion	1	0.2
14	cocaine	multiple	0, 10	chronic	NA	14	NA	antidepressant/anxiolytic	rat	male	go/go	reward/punisher	proportion	1	0.2
	d-amphetamine	multiple	0, 2	chronic	NA	14	NA	antidepressant/anxiolytic	rat	male	go/go	reward/punisher	proportion	1	0.2
15	cocaine	multiple	0, 1, 2, 5	acute	30	NA	NA	antidepressant/anxiolytic	rat	male	go/go	reward/punisher	proportion	1	0.2
	mazindol	multiple	0, 0.5, 1 2	acute	30	NA	NA	antidepressant/anxiolytic	rat	male	go/go	reward/punisher	proportion	1	0.2
16	phencyclidine	multiple	0, 2	chronic (wash-out)	NA	NA	8, 9, 10, 11, 12	depressant/anxiogenic	rat	female	go/go	reward/reward	proportion	1	0.5
17	p-Chlorophenylalanine	serotinergic system	0, 50	chronic (wash-out)	NA	NA	1,2,3,8,9,10	depressant/anxiogenic	pig	female	go/no-go	reward/punisher	latency	3	0.14
18	tryptophan	serotinergic system	NA	chronic	NA	6,7,8,13,14,15	NA	antidepressant/anxiolytic	pig	female	go/no-go	reward/punisher	latency	3	0.14
19	ghrelin	multiple	0, 0.007	acute	10	NA	NA	depressant/anxiogenic	sheep	female	go/no-go	reward/punisher	proportion	3	0.6
20	morphine	opioid system	0, 1	acute	10	NA	NA	antidepressant/anxiolytic	sheep	female	go/no-go	reward/punisher	latency	3	0.6
	naloxone	opioid system	0, 2	acute	10	NA	NA	depressant/anxiogenic	sheep	female	go/no-go	reward/punisher	latency	3	0.6

### 2.5 Effect size and sampling variance calculation

As latency data are bounded at zero and proportion data are bounded between zero and one data obtained from the judgement bias task do not follow a Gaussian or normal distribution. The delta method (Taylor approximation) was used to adjust the extracted mean ($\bar{x}$) and (sampling) variance (*sd*^2^) prior to calculating the effect size to account for the non-normality of the raw data (Nakagawa et al., 2017a). For extracted latency data, which were assumed to follow a log-normal distribution, this adjustment was calculated via the log transformation as:

(1) $$\bar{\ln(x)}=\ln(\bar{x})-\ln\left(\sqrt{1+\frac{{\mathrm{sd}}^{2}}{{\bar{x}}^{2}}}\right)$$

(2) $${\mathrm{sd}}_{\ln}^{2}=\ln\left(1+\frac{{\mathrm{sd}}^{2}}{{\bar{x}}^{2}}\right)$$

In this case, the transformed sampling variance is exact and not an approximation.

For extracted proportion data, which were assumed to follow a binomial distribution, this adjustment was calculated via the logit transformation as (Nakagawa and Schielzeth, 2010):

(3) $$\bar{\mathrm{logit}(x)}=\mathrm{logit}(\bar{x})+\frac{{\mathrm{sd}}^{2}}{2}\left(\frac{1}{{\left(1-\bar{x}\right)}^{2}}-\frac{1}{{\bar{x}}^{2}}\right)$$

(4) $${\mathrm{sd}}_{\mathrm{logit}}^{2}={\mathrm{sd}}^{2}{\left(\frac{1}{\bar{x}}+\frac{1}{1-\bar{x}}\right)}^{2}$$

Hedge's g (1981), a measure of effect size based standardized differences in means, was then calculated as the difference between the means of the relatively positive treatment (in which a relatively more positive affective state was expected, as outlined above) ${\bar{x}}_{+\mathrm{ve}}$ and means of the relatively negative treatment (in which a relatively less positive affective state was expected, as outlined above) ${\bar{x}}_{-\mathrm{ve}}$, divided by the pooled standard deviation, *sd*_*pool*_, and then adjusted for biases arising from small sample sizes by factor *J* which depended on the sample size of the relatively positive *n*_+*ve*_ and relatively negative *n*_−*ve*_ groups:

(5) $$\mathrm{SMD}=\frac{{\bar{x}}_{+\mathrm{ve}}-{\bar{x}}_{-\mathrm{ve}}}{{\mathrm{sd}}_{\mathrm{pool}}}\dot{}J$$

(6) $${\mathrm{sd}}_{\mathrm{pool}}=\sqrt{\frac{(n_{+\mathrm{ve}}-1){\mathrm{sd}}_{+\mathrm{ve}}^{2}+(n_{-\mathrm{ve}}-1){\mathrm{sd}}_{-\mathrm{ve}}^{2}}{n_{+\mathrm{ve}}+n_{-\mathrm{ve}}-2}}$$

(7) $$J=\left(1-\frac{3}{4(n_{+\mathrm{ve}}+n_{-\mathrm{ve}})-9}\right)$$

For the latency data, Hedge's g was multiplied by minus one to account for a higher proportion being equivalent to a lower latency, in terms of judgement bias.

The sampling variance was calculated as follows:

(8) $${\mathrm{se}}_{\mathrm{SMD}}^{2}=\frac{n_{+\mathrm{ve}}+n_{-\mathrm{ve}}}{n_{+\mathrm{ve}}n_{-\mathrm{ve}}}+\frac{{\mathrm{SMD}}^{2}}{2(n_{+\mathrm{ve}}+n_{-\mathrm{ve}})}$$

To account for shared controls, if one vehicle treatment group was compared to multiple drug treatment groups, an additional effect size and sampling variance was calculated based on a sample size for the vehicle group that had been divided by the number of treatment groups (Higgins and Green, 2011).

### 2.6 Meta-analysis and meta-regression models

The meta-analysis and meta-regression were conducted using the function, rma.mv from the R (R Core Team, 2017) package metafor (Viechtbauer, 2010); this function allowed us to fit multilevel meta-analytic and meta-regression models (Nakagawa et al., 2017b). All models included drug, institution at which the research was conducted, and effect ID (a unique ID given to each effect size) as random effects to account for the non-independence of effect sizes from studies conducted at the same institute or using the same drug (Van den Noortgate et al., 2013), and were fit using restricted maximum likelihood. The Knapp and Hartung adjustment was applied to all analyses (Knapp and Hartung, 2003). Initially, an intercept only model was fit to the effect sizes. A *p*-value for this model was obtained using a Wald-type test based on a *t*-distribution. Heterogeneity was assessed by calculating the *I*^2^ values for each random effect in the model and an overall *I*^2^ value for the model, following (Nakagawa and Santos, 2012), which is an extension of the original *I*^2^ (Higgins and Thompson, 2002).

Meta-regression was used to examine whether the following categorical and continuous moderators significantly contributed to variation between effect sizes: the dosing duration (chronic, acute, or chronic wash-out), the time between administration and testing (acute studies only), the number of days since the first dose (chronic studies only), the dosage differences between treatments from which the effect size was calculated, the neurobiological target of the drug, the manipulation type (positive or negative affect induction), the species tested, and the outcome variable used (latency or proportion), presented cue (positive reference cue, near-positive probe cue, midpoint probe cue, near-negative probe cue, negative reference cue), sex of the experimental subjects (all male, all female, or both male and female), reinforcement type (reward-punishment, reward-null, or reward-reward), response type (go/no-go, or go/go), cue type (reference or probe), and proportion of probe cues to reference cues in the test session. An omnibus test based on an *F* distribution, which examines the degree of variance explained by a moderator, was used to assess the significance of each moderator (Viechtbauer, 2010). To further investigate significant moderators, pairwise comparisons were made between the mean effect size for each level of the moderator. A Wald-type test was used to assess the significance of these pairwise comparisons. Moderators which were significant in the meta-regression were subsequently included together in a full model and their influence on the effect sizes was re-assessed. To verify that the model of best fit included all moderators, Akaike's information criterion (AIC) was calculated for the full model and was compared to models where a moderator had been removed.

### 2.7 Subset analyses

**Fig. 2:**
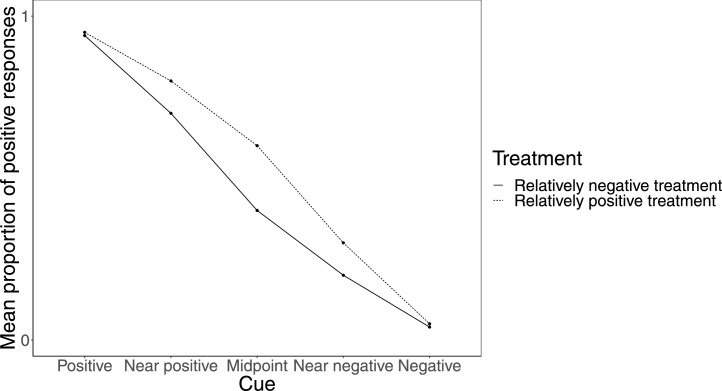
Example of hypothesised data from the judgement bias task with two treatments; one designed to induce a relatively positive affective state (relatively favourable treatment) and another designed to induce a relatively negative affective state (relatively unfavourable treatment). While the mean proportion of positive responses is almost identical at the positive and negative reference cue, a treatment difference is observed at the probe cues.

As affect is hypothesised to exert a greater influence on decision-making under ambiguity than under certainty, any treatment designed to pharmacologically induce a neurobiological state associated with a relatively more positive or negative affective state is expected to have the greatest influence on judgement bias at the ambiguous probe cues (see Fig. 2 for example of hypothesised data) (Mendl et al., 2009, Mendl et al., 2010). There are also methodological and theoretical reasons as to why an effect may be observed at one cue and not others. For example, a cue may be too perceptually similar to either of the reference cues for there to be ambiguity about the outcome, or a potential punisher may be much more aversive than the reward is rewarding, to the extent that all animals will avoid probe cues that are similar to the negative reference cue. By considering all cues equally (including reference cues), the effect of an affective manipulation might be obscured, potentially leading to the false inference of no significant effect. To this end, we conducted an additional analysis on a subset of data that included only the effect sizes from the probe cue with the largest absolute effect size for each drug within an article. Additionally, we analysed a second subset of data that included only the effect sizes for the cue with the absolute largest effect size in the direction of the mean effect size for each drug within an article to avoid including outlying effects that might not necessarily reflect the influence of the manipulation. If only one probe cue was presented in a study, data from this probe cue were included in the subset data.

### 2.8 Publication bias and sensitivity analysis

To assess the reliability of results across different analytical approaches and to check for a publication bias, the intercept-only and full meta-regression model were re-fit to the data under a Bayesian statistical framework using the R package MCMCglmm (Hadfield, 2010). The non-independence of effect sizes can also be accounted for using Bayesian methods. A parameter-expanded prior, allowing variance components to have different prior distributions, was used for both the random effect of drug and institution ID, while the prior variance for random effect of effect ID was fixed at one. Model fitting had 110,000 iterations, 10,000 burn-in periods, and thinning by every 100, resulting in an effective sample size of 1000. The result of this intercept-only model was compared to our initial intercept-only model. The ‘meta-analytic residuals’ (*sensu* (Nakagawa and Santos, 2012)) from full meta-regression model conducted in MCMCglmm were used to produce a funnel plot and run Egger's regression, which here regresses the meta-analytic residuals against precision (Egger et al., 1997, Nakagawa and Santos, 2012), and hence checks for a publication bias. Additionally, the intercept-only meta-analysis was repeated but with the effect size and sampling variance that had been adjusted (via the sample size) for shared controls, to assess whether this altered the results.

## 3 Results

### 3.1 Data review

We extracted 557 effect sizes from 20 articles that had been published by authors based at 10 different institutions (see Table 1, Table 2). Twenty-seven different drugs were used across these studies. The majority (328) of the effect sizes came from studies that had used drugs expected to induce a relatively positive affective state (anxiolytics or antidepressants, 12 articles), while the remainder used anxiogenic or depressant drugs (112 effect sizes, 9 articles). There were 408 effect sizes (14 articles) that came from studies using acute pharmacological manipulations, 97 effect sizes (6 articles) from studies using chronic pharmacological manipulations, and 52 effect sizes (5 articles) that came from the wash-out period of a chronic pharmacological manipulation. Most effect sizes came from studies using drugs that targeted the serotonergic system (198 effect sizes, 7 articles) while a high proportion of studies also used drugs that targeted a range of neurobiological systems (190 effect sizes, 9 articles) which included drugs such as cocaine and d-amphetamine which target the dopaminergic, serotoninergic, and adrenal systems. The remaining effect sizes were from experiments using drugs that specifically targeted GABAergic system (46 effect sizes, 4 articles), adrenergic system (43 effect sizes, 3 articles), dopaminergic system (36 effect sizes, 1 article), opioid system (20 effect sizes, 1 article), glucocorticoid system (15 effect sizes, 1 article), oxytocin system (9 effect sizes, 2 articles). Five different species were used across the studies; the most frequently used species according to the number of effect sizes was rat (418 effect sizes, 11 articles), followed by pig (60 effect sizes, 2 articles), sheep (50 effect sizes, 4 articles), chicken (23 effect sizes, 2 articles), and dog (6 effect sizes, 1 article). Proportion was more commonly used as the outcome measure (435 effect sizes, 12 articles) compared with latency (122 effect sizes, 8 articles). The majority of effect sizes came from studies using only male subjects (421 effect sizes, 11 articles), followed by only female subjects (130 effect sizes, 8 articles), and six effect sizes (1 articles) came from studies that used both male and female subjects. The most common reinforcement type was reward-punisher (420 effect sizes, 16 articles), followed by reward-reward reinforcement (131 effect sizes, 3 articles), and reward-null (6 effect sizes, 1 article). There were more effect sizes from studies using a ‘go/go’ design (415 effect sizes, 10 articles) compared with a ‘go/no-go’ design (142 effect sizes, 10 articles).

Across the articles from the acute studies, the average time between the administration of the drug and testing was 32.903 ± 5.530 (mean ± SE) minutes. The average number of days between the start of the chronic drug treatment and testing was 9.000 ± 1.074, and the average days the animal had been withdrawn from a drug when tested in the wash-out period was 6.938 ± 0.824. The mean proportion of probe cues to reference cues used during a test session was 0.341 ± 0.037. There were 11 articles that used more than one probe cue and three of these articles examined the effect of more than one drug. In total, there were 14 sets of effect sizes obtained from different articles using different drugs which used more than one probe cue. The probe cue with the greatest absolute effect size was the near-positive probe cue on nine occasions, the near-negative probe cue on four occasions, and the midpoint probe cue on one occasion. The probe cue with the greatest absolute effect size was also the presented cue with the greatest absolute effect size in the direction of the mean effect for all but one of the sets of effect sizes, where the near-positive probe cue had the greatest absolute effect sizes and the near-negative probe cue had the greatest absolute effect size in the direction of the mean effect.

### 3.2 Meta-analysis

Overall, considering all effect sizes equally, affect-altering drugs did not significantly induce a judgement bias in non-human animals, although a small effect size (*sensu* (Cohen, 1988): small = 0.20, moderate = 0.5, large = 0.8) was observed (mean = 0.239, 95% confidence interval or CI = −0.047–0.525, *t*_556_ = 1.639, *p* = 0.102). However, this needs to be interpreted in the context of the observed high total heterogeneity in the model, with an *I*^2^ value of 89.535 (>75% = high, (Higgins and Thompson, 2002)), indicative of wide variation in the extent to which pharmacological manipulations alter judgement bias that warrants further examination. The between-effect-size effect (i.e., residuals; 42.378%) and the between-drug effect (i.e., which drug were used; 35.112%) explained a large percentage of this heterogeneity, while a smaller percentage of variability was due to institutional variation (12.044%). Heterogeneity between effect sizes was further explored through the meta-regression.

### 3.3 Subset analyses

However, as aforementioned, given the theoretical framework for judgement bias, we did not anticipate that effect sizes would be equal across all cues. Instead, we considered it likely that pharmacological manipulations would exert the greatest influence at only one of the probe cues, with proximity of this cue to the reference cues differing between studies as a result of different methodologies. Hence, subset analyses were conducted to assess the extent to which the pharmacological manipulations of affect altered judgement bias at, at least, one of the probe cues. Pharmacological manipulations using drugs which have affect-altering properties were found to have a significant small to moderate effect on judgement bias when the analysis was repeated on the subset data comprising only data from the ambiguous cue with the largest absolute effect size (mean = 0.394, CI = −0.046–0.7270.017–0.770, *t*_154_ = 2.067, *p* = 0.040), and a significant small to moderate effect on the subset data comprising only data from the ambiguous cue with the largest absolute effect size in the direction of the mean effect (mean = 0.455, CI = 0.061–0.849, *t*_154_ = 2.279, *p* = 0.024).

### 3.4 Meta-regression

**Fig. 3:**
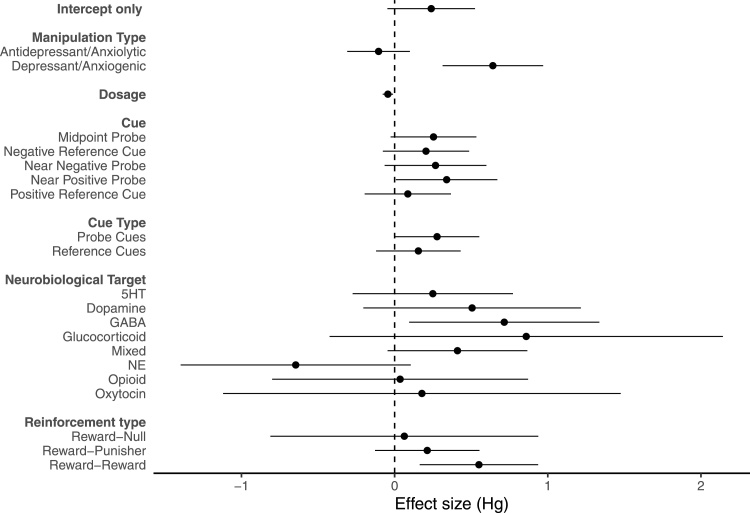
Forest plot with a meta-analytic mean (intercept-only model) and significant moderators from univariate meta-regression models. Each point represents the mean effect size for each moderator and error bars represent the 95% confidence interval.

The meta-regression revealed that several moderators significantly explained the observed heterogeneity among the extracted effect sizes, these moderators were: pharmacological manipulation type (Fig. 3: *F*_(1,555)_ = 16.056, *p* < 0.001), dosage (*F*_(1,519)_ = 6.614, *p* = 0.010), the reinforcement type (*F*_(2,554)_ = 3.653, *p* = 0.027) and the cue type (*F*_(1,555)_ = 4.725, *p* = 0.030). The presented cue (*F*_(4,552)_ = 2.002, *p* = 0.093), and the neurobiological target of the drug were marginally non-significant moderators (*F*_(8,548)_ = 1.835, *p* = 0.079). More specifically, pharmacological manipulations expected to induce a relatively negative affective state (either depressant or anxiogenic) had a greater effect on judgement bias than those expected to induce a relatively positive affective state (Table 3). Greater differences in dosage between the relatively positive and negative treatments were associated with smaller effect sizes. The greatest effect size was found when the reinforcement used for the reference cues was a high reward and low reward, compared with a reward and punisher. The effect of the pharmacological manipulation was greater at the probe cues compared with the reference cues (Table 3). The effect of the pharmacological manipulation was weaker at the positive reference cue compared to the midpoint probe cue, and near-positive probe cue, and tended to be weaker than the negative reference cue (Table 3). There was no difference in effect size at the positive reference cue compared with the near-negative reference cue (Table 3). The remaining moderators tested were not found to significantly explain variation in effect size. The effect of drugs targeting the adrenergic system differed significantly from all other drugs used apart from drugs targeting the opioid and oxytocin system (Table 3). Drugs targeting the adrenergic system had the opposite effect than expected; a negative judgement bias was induced when a positive judgement bias was hypothesised. Other moderators with non-significant effects included: species (*F*_(4,552)_ = 0.835, *p* = 0.503), dosing frequency (*F*_(2,554)_ = 0.108, *p* = 0.898), time since last dose (*F*_(1,406)_ = 0.467, *p* = 0.495), number of days since first treatment (*F*_(1,95)_ = 1.169, *p* = 0.282), sex (*F*_(2,554)_ = 0.328, *p* = 0.720), response type (*F*_(1,555)_ = 0.040, *p* = 0.842), proportion of ambiguous cues to reference cues (*F*_(1,555)_ = 1.531, *p* = 0.217), and outcome measure (*F*_(1,555)_ = 0.139, *p* = 0.709).

**Table 3:** Pairwise comparison of each level of significant moderators from the meta-regression.

Variable	Model	Mean difference	CI lower bound	CI upper bound	*p*-Value
*Cue*	**Midpoint – Positive**	**0.168**	**0.028**	**0.307**	**0.019**
	Negative – Positive	0.119	−0.022	0.26	0.099
	Near Negative – Positive	0.181	−0.059	0.421	0.140
	**Near Positive – Positive**	**0.253**	**0.015**	**0.492**	**0.037**
	Midpoint – Near Positive	−0.086	−0.324	0.153	0.480
	Negative – Near Positive	−0.135	−0.375	0.105	0.270
	Near Negative – Near Positive	−0.073	−0.357	0.212	0.616
	Negative – Midpoint	−0.049	−0.19	0.091	0.493
	Near Negative – Midpoint	0.013	−0.227	0.253	0.914
	Negative – Near Negative	−0.062	−0.304	0.179	0.612
*Cue type*	**Reference – Probe**	**−0.122**	**−0.232**	**−0.012**	**0.030**
*Manipulation Type*	**Depressant/Anxiogenic – Antidepressant/Anxiolytic**	**0.746**	**0.38**	**1.112**	<**0.001**
*Neurobiological Target*	**Serotoninergic – Adrenergic**	**0.895**	**0.118**	**1.672**	**0.024**
	**Dopaminergic – Adrenergic**	**1.152**	**0.254**	**2.049**	**0.012**
	**GABAergic – Adrenergic**	**1.362**	**0.54**	**2.184**	**0.001**
	**Glucocorticoid – Adrenergic**	**1.505**	**0.017**	**2.993**	**0.047**
	**Multiple – Adrenergic**	**1.056**	**0.346**	**1.766**	**0.004**
	Opioid – Adrenergic	0.682	−0.365	1.729	0.201
	Oxytocin – Adrenergic	0.824	−0.676	2.324	0.281
	Serotoninergic – Multiple	−0.161	−0.622	0.3	0.493
	Dopaminergic – Multiple	0.096	−0.533	0.724	0.766
	GABAergic – Multiple	0.306	−0.244	0.855	0.276
	Glucocorticoid – Multiple	0.499	−0.914	1.812	0.518
	Opioid – Multiple	−0.374	−1.208	0.459	0.378
	Oxytocin – Multiple	−0.232	−1.608	1.144	0.741
	Dopaminergic – Serotoninergic	0.256	−0.435	0.948	0.467
	GABAergic – Serotoninergic	0.466	−0.156	1.088	0.141
	Glucocorticoid – Serotoninergic	0.61	−0.777	1.996	0.388
	Opioid – Serotoninergic	−0.214	−1.035	0.608	0.610
	Oxytocin – Serotoninergic	−0.071	−1.471	1.328	0.920
	GABAergic – Dopaminergic	0.21	−0.542	0.962	0.584
	Glucocorticoid – Dopaminergic	0.353	−1.114	1.821	0.636
	Opioid – Dopaminergic	−0.47	−1.469	0.529	0.356
	Oxytocin – Dopaminergic	−0.328	−1.807	1.152	0.664
	Glucocorticoid – GABAergic	0.143	−1.283	1.570	0.843
	Opioid – GABAergic	−0.68	−1.626	0.266	0.159
	Oxytocin – GABAergic	−0.538	−1.976	0.901	0.463
	Opioid – Glucocorticoid	−0.823	−2.355	0.708	0.292
	Oxytocin – Glucocorticoid	−0.681	−2.507	1.145	0.464
	Oxytocin – Opioid	0.142	−1.401	1.686	0.856
*Reinforcement Type*	Reward/Punisher – Reward/Null	0.15	−0.702	1.002	0.730
	Reward/Reward – Reward/Null	0.487	−0.395	1.369	0.279
	**Reward/Reward – Reward/Punisher**	**0.337**	**0.089**	**0.585**	**0.008**

Bold signifies that the model was significant at *p* < 0.05.

The best fitting model included cue (i.e. positive reference cue, midpoint probe cue, negative reference cue, near negative probe cue and near positive probe cue) instead of cue type (i.e. reference or probe) (ΔAIC (i.e. difference in AIC values between models) = 0.437), and all significant moderators identified in the univariate meta-regression. Removal of the neurobiological target of the drug (ΔAIC = 13.511), dosage (ΔAIC = 6.705), cue (ΔAIC = 3.583), reinforcement type (ΔAIC = 7.573), and manipulation type (ΔAIC = 6.465) resulted in a poorer fit according to the AIC values. The best fitting model had a marginal *R*^2^ value (*sensu* (Nakagawa and Schielzeth, 2013)) of 72.844%. In this model, the difference between effect sizes where a relatively positive compared with relatively negative affective state had been induced was significant, with a moderate effect size (Δmean = 0.582, CI = 0.054–1.110, *t*_506_ = 2.164, *p* = 0.031). Effect sizes from drugs targeting the adrenergic system were overall in the opposite direction to expected and there was a large and significant difference in effect sizes between adrenergic system targeting drugs and multiple system targeting drugs (Δmean = 0.852, CI = 0.043–1.661, *t*_507_ = 2.069, *p* = 0.039) and GABAergic system targeting drugs (Δmean = 1.299, CI = 0.369–2.228, *t*_507_ = 2.746, *p* = 0.006), and a large but marginally non-significant difference in effect sizes between adrenergic system targeting drugs and serotonergic system targeting drugs (Δmean = 0.817, CI = −0.073–1.707, *t*_507_ = 1.803, *p* = 0.072), dopaminergic system targeting drugs (Δmean = 0.936, CI = −0.083–1.956, *t*_507_ = 1.804, *p* = 0.072), glucocorticoid system targeting drugs (Δmean = 1.451, CI = −0.250–3.151, *t*_507_ = 1.676, *p* = 0.094). There was a large but non-significant difference between the effect sizes of drugs targeting the adrenergic and oxytocin system (Δmean = 0.961, CI = −0.894–2.815, *t*_507_ = 1.018, *p* = 0.309) and moderate but non-significant difference between the effect sizes of drugs targeting the adrenergic compared with opioid targeting drugs (Δmean = 0.555, CI = −0.621–1.732, *t*_507_ = 0.927, *p* = 0.354). Effect sizes were significantly weaker at the positive reference cue compared with the midpoint probe cue (Δmean = 0.163, CI = 0.019–0.308, *t*_507_ = 2.218, *p* = 0.027), and near-positive probe cue (Δmean = 0.289, CI = 0.025–0.553, *t*_507_ = 2.148, *p* = 0.032). Effect sizes at the positive reference cue were not significantly different from effect sizes at the negative reference cue (Δmean = 0.118, CI = −0.028–0.264, *t*_507_ = 1.592, *p* = 0.112) or at the near-negative probe cue (Δmean = 0.206, CI = −0.060–0.472, *t*_507_ = 1.519, *p* = 0.129). There was a small and significant difference between effect sizes from studies using high and low rewards as the more and less favourable outcome, respectively, compared with studies which used rewards and punishers (Δmean =−0.366, CI = −0.626–0.106, *t*_543_ =−2.764, *p* = 0.006), while there was a moderate but non-significant difference between studies that used high and low rewards and those that used a reward and null outcome (Δmean =−0.546, CI = −1.548–0.457, *t*_543_ =−1.070, *p* = 0.285).

### 3.5 Study exclusion

As the initial analysis revealed that drugs targeting the adrenergic system had the opposite effect on judgement bias than hypothesised, which differed significantly from the majority of the other drugs not specifically targeting the adrenergic system demonstrating that these drugs produced an anomalous effect in comparison to all other drugs examined, we re-assessed the rationale for classification of adrenergic-targeting drugs as either anxiolytic/antidepressant or anxiogenic/depressant. There is conflicting evidence about the affect altering properties of adrenergic system targeting drugs in both non-human (Tanaka et al., 2000, Inoue et al., 2006) and human (Harmer et al., 2008) animals. Moreover, studies have demonstrated that acute negative affect is associated with increased levels of norepinephrine, while chronic negative affect is associated with decreased levels of norepinephrine (Morilak et al., 2005, Roth et al., 1982), which further complicates assessment of whether the neurobiological state induced by the pharmacological manipulations most resembled that of a relatively negative or positive affective state. Consequently, we made the post-hoc decision to re-analyse the data excluding effect sizes from studies using adrenergic-system targeting drugs. Three studies had used adrenergic-system targeting drugs; one study had used clonidine and the other two studies had used reboxetine. Both clonidine and reboxetine are considered to induce a relatively positive affective state. These studies accounted for 7.719% (43) of the effect sizes analysed.

### 3.6 Post-exclusion meta-analysis

Following the exclusion of these effect sizes, a moderate overall effect was observed; pharmacological manipulations which induced a neurobiological state associated with relatively more positive or negative affect were found to significantly influence judgement bias in the predicted direction (mean = 0.400, CI = 0.056–0.744, *t*_513_ = 2.287, *p* = 0.023). However, there again existed high heterogeneity (*I*^2^ = 89.746%); with 38.362% attributable to between-effect-size effects, 20.732% to between-drug effects, and 30.653% to institutional variation. The meta-analysis using both data subsets (using only one probe cue) revealed a significant and moderate overall effect of pharmacological manipulations on judgement bias (absolute greatest probe cue effect sizes: mean = 0.520, CI = 0.116–0.924, *t*_144_ = 2.543, *p* = 0.012; and absolute greatest probe cue effect in direction of mean: mean = 0.579, CI = 0.157–1.001, *t*_144_ = 2.711, *p* = 0.008).

### 3.7 Post-exclusion meta-regression

**Fig. 4:**
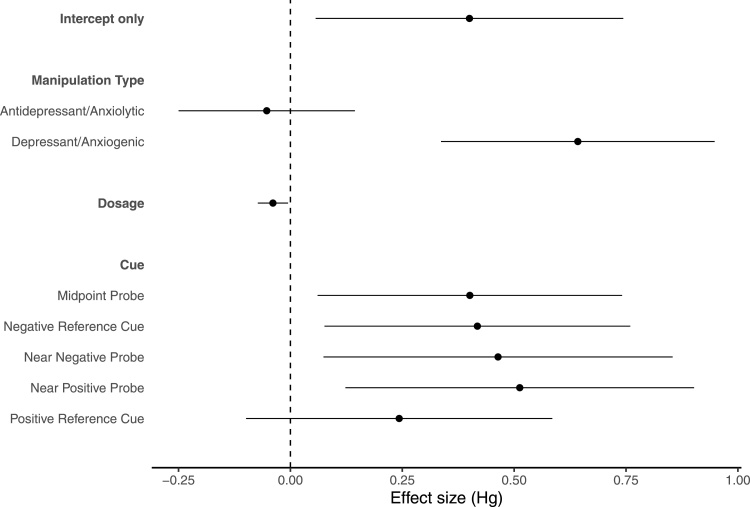
Forest plot with a meta-analytic mean (intercept-only model) and significant moderators from univariate meta-regression models following the exclusion of adrenergic system targeting drugs. Each point represents the mean effect size for each moderator and error bars represent the 95% confidence interval.

While manipulation type (Fig. 4: *F*_(1,512)_ = 15.700, *p* < 0.001) and dose (Fig. 4: *F*_(1,476)_ = 5.169, *p* = 0.023), remained significant as moderators when studies using adrenergic system targeting drugs were excluded, the presented cue (Fig. 4: *F*_(4,509)_ = 2.396, *p* = 0.049) was now significant as opposed to marginally non-significant and drug target (*F*_(6,506)_ = 0.578, *p* = 0.748) cue type (*F*_(1,512)_ = 2.594, *p* = 0.108), and reinforcement type (*F*_(2,511)_ = 0.144, *p* = 0.866) were no longer significant. The model which included all three significant moderators provided a better fit than the models which excluded manipulation type (ΔAIC = 13.465), cue (ΔAIC = 2.299), and dose (ΔAIC = 5.430). This full model had a marginal *R*^2^ value of 61.008%.

The difference between effect sizes at the midpoint probe cue and positive reference cue was very small but significant (Δmean = 0.154, CI = 0.011–0.297, *t*_471_ = 2.111, *p* = 0.035, and the difference between effect sizes at the positive reference cue and near-positive probe cue was small but marginally non-significant (Δmean = 0.245, CI = −0.035–0.525, *t*_471_ = 1.716, *p* = 0.087). Contrary to the previous analysis including adrenergic-targeting drugs, a very small but significant difference was found between effect sizes at the negative and positive reference cues, with greater effect sizes at the negative reference cue (Δmean = 0.183, CI = 0.039–0.327, *t*_471_ = 2.491, *p* = 0.013). The difference in effect sizes between the positive reference and near-negative probe cue remained non-significant (Δmean = 0.192, CI = −0.089–0.474, *t*_471_ = 1.341, *p* = 0.181). Effect sizes were still observed to be greater when the anxiogenic or depressant drugs were used compared to the antidepressant or anxiolytic drugs with a moderate difference in effect sizes (Δmean = 0.701, CI = 0.348–1.055, *t*_471_ = 3.897, *p* < 0.001), and effect sizes remained significantly greater when there were smaller differences in dosage between the relatively positive and relatively negative treatment, although the effect was very small (mean = −0.0379, CI = −0.071–0.004, *t*_471_ =−2.223, *p* = 0.027).

### 3.8 Publication bias and sensitivity analysis

The results of the Bayesian meta-analysis were consistent with the results of our likelihood-based meta-analyses both prior to and following the removal of effect sizes from studies using drugs targeting the adrenergic system. The effect of the pharmacological manipulations on judgement bias was not significant prior to data exclusion (mean = 0.242, 95% credible interval = −0.097–0.666, *p* = 0.194), but a marginally non-significant overall effect emerged following data exclusion from studies using adrenergic-system targeting drugs (mean = 0.387, credible interval = 0.020–0.864, *p* = 0.056).

**Fig. 5:**
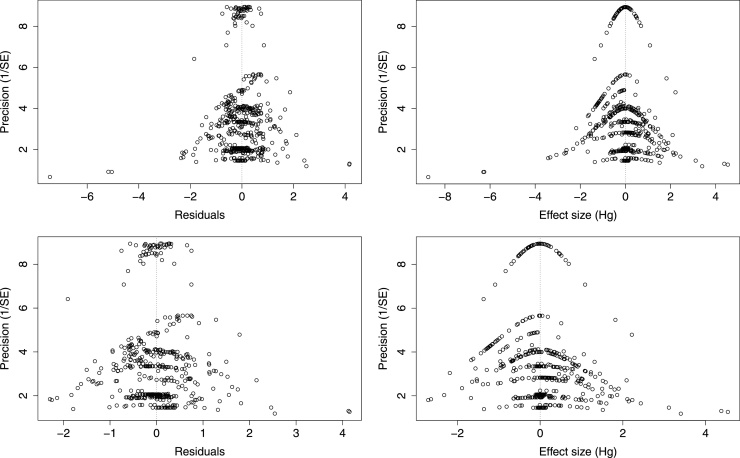
Funnel plots of (a) the meta-analytic residual values (residuals + sampling errors) for the full meta-regression model prior to exclusion of effect sizes from studies using adrenergic system targeting drugs; (b) the raw effect sizes and the inverse standard errors prior to exclusion of effect sizes from studies using adrenergic system targeting drugs; (c) the meta-analytic residual values for the full meta-regression model following exclusion of effect sizes from studies using adrenergic system targeting drugs; (d) the raw effect sizes and the inverse standard errors following exclusion of effect sizes from studies using adrenergic system targeting drugs.

Visual inspection of the funnel plots produced from the meta-analytic residuals and raw effect sizes (Fig. 5) did not indicate that a publication bias was present, nor did the results of Egger's test on either the analysis prior to (*t*_519_ =−0.419, *p* = 0.675) or following (*t*_476_ = 0.568, *p* = 0.570) the exclusion of data.

Re-analysis of the intercept-only model using the effect sizes and variances that had been adjusted for shared controls did not alter the results qualitatively. The result prior to data exclusion was statistically non-significant (mean = 0.240, CI = −0.047–0.527, *t*_522_ = 1.641, *p* = 0.101) while following data exclusion was significant (mean = 0.401, CI = 0.057–0.745, *t*_476_ = 2.291, *p* = 0.022).

## 4 Discussion

Judgement bias is a relatively new and promising measure of animal affect that may provide a useful alternative to more common behavioural assays used to assess the efficacy of potential pharmacological treatments of mood disorders, such as the forced swim test. Empirical studies with human subjects have supported its construct validity (Nygren et al., 1996, Johnson and Tversky, 1983, Wright and Bower, 1992, Paul et al., 2011, Anderson et al., 2012). To examine its predictive validity, we conducted a systematic review and meta-analysis of studies investigating the effect of affect-altering drugs on judgement bias in non-human animals. We analysed data from 20 published research articles which yielded 557 effect sizes.

There was high heterogeneity (>75%) between the effect sizes observed (Senior et al., 2016) indicating strong variability in the extent to which pharmacological manipulations of affective state alter judgement bias. The drug used accounted for some of this variability, as did the institution at which the research was conducted, yet a high proportion of heterogeneity was also attributed to variation within drug and institution. Our meta-regression further highlighted a number of factors which explained variation in effect sizes including the neurobiological drug target, manipulation type (whether the drug was hypothesised to induce a negative or positive affective state), dosage, cue, and cue type (reference or probe).

Initially, considering all effect sizes across all cues equally (including references cues), we found no significant overall effect of affect-altering drugs on judgement bias in non-human animals. However, because there are theoretical and empirical reasons for an effect being more likely at ambiguous cues as opposed to reference cues, and/or to occur at one ambiguous cue but not others (e.g. because the others may happen to be too perceptually similar to the reference cues, see Methods), considering all cues equally may obscure an effect of a treatment manipulation. Indeed, judgement bias studies often report effects that are observed at only a subset of (ambiguous) cues (e.g. (Bethell and Koyama, 2015)). Consequently, we also carried out analyses using subsets of data that included only (i) effect sizes for the probe cue with the largest absolute effect size; (ii) effect sizes for the cue with the absolute largest effect size in the direction of the mean effect size. These analyses revealed that the pharmacological manipulations altered judgement bias in the predicted direction.

The results of the meta-regression showed a clear moderating effect of the neurobiological drug target, particularly of drugs targeting the adrenergic system, whose effect differed significantly from the majority of other drugs used. A small to medium effect using data from all cues was found following the removal of data from studies targeting the adrenergic system, and a moderate effect was found when considering data from the subset analyses described above. Thus, this meta-analysis provides support for the validity of judgement bias as measure of affect in non-human animals, having demonstrated that pharmacological manipulations using drugs known to influence affect in humans overall alter judgement bias in non-human animals at the probe cues in the predicted direction. However, there exist a few caveats; it is important to state that this result should not be interpreted as evidence that the pharmacological manipulations did alter affect and that this shift in affect directly influenced judgement bias. Instead, this result demonstrates that pharmacologically-induced neurobiological states associated with relatively positive or negative affect alter judgement bias in the predicted direction, given hypotheses about how affect should alter judgement bias – judgement bias reliably predicted the affect-altering properties of the pharmacological manipulation. However, the pharmacological manipulations may have altered judgement bias through a variety of mechanisms and we cannot preclude the possibility that manipulations did not alter affect in non-human animals as they would in human subjects, or that the observed shift in judgement bias was directly attributable to a shift in affect.

The three excluded studies used either reboxetine, an antidepressant, or clonidine, which is used off-licence to treat anxiety disorders. Jointly, these drugs were found to exert an opposite effect on judgement bias; inducing a negative judgement bias when a positive judgement bias was predicted. Paradoxically, depression and anxiety are known side effects of these drugs (Center for Drug Evaluation and Research, 2018). Moreover, these studies both used an acute dose which may explain why their effects were not in the predicted direction. Both norepinephrine and cortisol increase in response to stress and acute dosing of drugs which simultaneously elevate levels of norepineprine and cortisol have been shown to result in stress-like changes in the neural response to negative stimuli in humans (Kukolja et al., 2008). It is therefore feasible that the acute delivery of adrenergic-system targeting antidepressant drugs induced a neurobiological state associated with relatively negative rather than a positive affective state which resulted in the relatively negative judgement bias observed. This potential explanation is further supported by studies that have observed anxiety-like states in rodents following the administration of similar adrenergic-system targeting drugs (Tanaka et al., 2000, Inoue et al., 2006).

An alternative explanation could be related to another side effect of adrenergic agonists that has been documented in human and non-human animal subjects; sedation (Buerkle and Yaksh, 1998, Center for Drug Evaluation and Research, 2018) A sedated animal may not have been able to fully partake in the experiment or have been considerably slower to respond, leading to seemingly risk-averse (deemed ‘pessimistic’ in the judgement bias literature) responses. This is perhaps further supported by the finding that clonidine and reboxetine led to a increased latency to respond to the positive reference cue, as described by the authors of the studies included in this meta-analysis. However, further studies would be required to reveal the extent to which the results from these two studies can be generalised to all adrenergic system targeting drugs.

Depressant and anxiogenic drugs, had a greater effect on measured judgement bias than antidepressant and anxiolytic drugs. This result may reflect an interaction between the drugs administered and affective states arising from the process of being tested, which may sometimes be negative in their own right (e.g. invasive administration of drugs, social isolation during testing, and potential delivery of an aversive decision outcome). These factors may have enhanced the neurobiological effect of the depressant and anxiogenic drugs, while dampening the neurobiological effect of the antidepressant and anxiolytic drugs. Notably, the potential negative affective state induced by testing may also explain the finding, prior to exclusion of adrenergic targeting drugs, that effect sizes were greater when only rewards were used as reinforcement, as opposed to both reward and punishers. Indeed, in humans there is evidence to suggest that some affect-altering recreational drugs intensify the affective state of an individual prior to consumption, or result in the exaggerated interpretation of emotional stimuli (Parrott et al., 2011, Foisy et al., 2007). With regards to the development of novel treatments for mood disorders such as depression and anxiety, this perhaps suggests that attention should be given to the potential effects of the testing procedure on affect, and that greater sample sizes may be required to provide sufficient power for an effect of the potential pharmacological treatment to be detected.

Another possible explanation for the moderating effect of manipulation type is that there are floor effects which limit the impact antidepressant and anxiolytic drugs may have on judgement bias. There will be a physical limit to how quickly an animal can approach a cue, and the control animals may already be performing at or close to this limit, meaning that the animals that had been given a antidepressant and anxiolytic drug could not respond any quicker. However, this explanation will only be relevant to studies measuring approach latency. Similarly, the smoke-detector principle states that individuals should be overly responsive to potential threats (Nesse, 2001); just as the cost of a smoke detector not detecting a fire is far greater than the cost of the smoke detector sounding an alarm when there is no fire, false positives are also optimal in the detection of predators. An individual may continue to appear relatively cautious even when in a more positive affective state because the cost of not avoiding punishers (i.e. potential death) is so high that it would be suboptimal for an individual to behave in a more risky manner (i.e. making the ‘optimistic’ response which could lead to a punisher, as opposed to the ‘pessimistic’ response which is the safe option) (Nesse, 2001, Nettle and Bateson, 2012).

Although this meta-analysis did not identify a difference in effect sizes between studies which analysed proportion or latency data, it is important to discuss these two outcome measures. Typically, both latency and proportion data can be collected from judgement bias studies and it is unclear what drives a researcher to select either measure. There are merits and disadvantages of using either measure; while latency contains more information than proportion data, in the sense that as a continuous variable it may identify variation that proportion data cannot, it may also be more subject to influences from other factors such as any effect of the drug on motor responses as outlined above or other cognitive biases such as attention biases (Mendl et al., 2009).

Greater effects were observed when there were relatively smaller differences in dosage between treatments. This is consistent with the inverted U-shaped dose-response function that is sometimes observed in drug studies (Calabrese and Baldwin, 2001a, Calabrese and Baldwin, 2001b, Calabrese and Baldwin, 2001c). This result may reflect that higher doses increase the probability of side effects which may interfere with task performance (Lazarou et al., 1998, Furukawa et al., 2002). Adverse effects that alter the motivation of the animal (e.g. reduced appetite), their consummatory behaviour (e.g. nausea), or psychomotor abilities (e.g. sedation) are likely to affect judgement bias. Such side effects are common to several affect-altering drugs (Center for Drug Evaluation and Research, 2018). It may therefore be sensible to take measures of activity or food consumption concurrent to the judgement bias task to assess the potential impact of side effects of drug manipulations.

The meta-analysis also found that the effects of the pharmacological manipulations on judgement bias were weakest at the positive reference cue, and that the effect of pharmacological manipulations was greater when only the probe cue with the greatest effect size within each drug and article were analysed. This reflects that pharmacological manipulations using affect-altering drugs exert a stronger influence on trials where there is ambiguity about the outcome of the trial compared with trials where the reward is certain. On presentation of the positive reference cue, there should be little ambiguity about the outcome, and it would be expected that the animal should make the response that allows them to obtain the reward on a high proportion of trials. The influence of any manipulation which putatively alters affect should be greatest when there is uncertainty about the outcome as subjective probabilities of uncertain outcomes are thought to be more strongly informed by an individual's affective state (Mendl et al., 2010, Mendl and Paul, 2019, Trimmer et al., 2013). Thus, this finding is consistent with the theoretical framework underlying judgement bias. However, given that a number of cognitive processes could lead to a shift in judgement bias at the probe but not the positive reference cue, this finding does not negate the possibility that cognitive processes other than probability estimation underlie the relationship between the pharmacological manipulations and decision-making on the judgement bias task.

It is unclear why the extracted effect sizes were not smaller at the negative reference cue following exclusion of effect sizes from studies that had used adrenergic-targeting drugs. The pharmacological manipulations were not expected to exert a similar effect at the negative reference cue compared with the probe cues, as there should be little uncertainty about the outcome when the reference cues are presented. Moreover, in studies in which multiple probe cues were presented, the pharmacological manipulations rarely exerted the greatest influence at midpoint cue, where there should be the greatest uncertainty about the outcome. This further suggests that pharmacologically-induced neurobiological states associated with relatively more positive or negative affect do not necessarily induce a greater judgement bias as uncertainty about the decision outcome increases. Both valuation and probability of decision outcomes have been identified as key components of decision-making that underlie variation in the judgement bias task (Iigaya et al., 2016); an individual might be more likely to make a risky or more ‘optimistic’ response if they considered the reward to more probable or punisher to be less probable or if they considered the reward to be more valuable or punisher to be less aversive (Mendl et al., 2009, Rangel et al., 2008). Speculatively, it is possible that the pharmacological manipulations altered the valuation of the punisher, hence altering responses to its presentation. A better understanding of the cognitive processes underlying judgement bias and how they relate to affect, which could be achieved through a battery of tests or computational modelling of judgement bias data, would be highly valuable. Similarly, it is possible that some findings reflect differences between the reward and punisher systems. Indeed, prior to the removal of adrenergic drugs from the dataset, we found that the effect of the pharmacological manipulation was greater when no reward was used as the negative outcome compared to when a punisher was used as the negative outcome.

Our meta-analysis found no evidence to indicate that the species used, the dosing frequency, the time since last dose in acute studies, the number of days since first treatment in chronic studies, the outcome variable used, the biological sex of the individuals studied, the reinforcement type, or response type had moderating effects on the influence of pharmacological manipulations of affect on judgement bias. While this might reflect that there is insufficient power to detect an effect, it might indicate that judgement bias is robust to variation in methodology and across species. Interestingly, despite being one of the most commonly used non-human animal species in research, none of the studies included in this meta-analysis used mice (Home Office, 2017, National Research Council, 1988). As judgement bias tasks have been successfully conducted in mice (Hintze et al., 2018, Krakenberg et al., 2019), we consider that it would be highly worthwhile to examine the extent to which pharmacological manipulations alter judgement bias in mice. We found no evidence to suggest a publication bias.

Future studies should attempt to account for the potential side effects of pharmacological manipulations. Observing behaviour following drug administration, for example activity levels and food and water consumption, may help to highlight potential adverse effects. The majority of effect sizes extracted in this meta-analysis were from studies using serotonergic-system targeting drugs. While this is unsurprising given that commonly prescribed antidepressants target the serotonin system (Mars et al., 2017), mood disorders are associated with dysfunction in several neurological systems and further investigation of the influence of pharmacologically-induced changes in the activity of these systems may be beneficial (Nemeroff and Owens, 2002, Nestler et al., 2002, Joëls and Baram, 2009). This meta-analysis has highlighted that multiple probe cues may be preferable in future studies. Pharmacological manipulations using affect-altering drugs do not necessarily exert the strongest influence of judgement bias at the most ambiguous cue, as found in this meta-analysis, and using multiple cues would allow a more comprehensive assessment of the effect of the manipulation. Finally, it would be worthwhile to assess the efficacy of judgement bias as a measure of pharmacological manipulations of affect in mice.

## 5 Conclusion

To conclude, this meta-analysis has provided evidence that judgement bias has predictive validity as a measure of pharmacologically-induced neurobiological states associated with relatively negative or positive affect, which supports judgement bias as a measure of affect in non-human animals. A key issue identified in this study is the potential interference of drug side effects on judgement bias. In particular, the contrary effect of adrenergic-targeting affect-altering drugs and the greater effect of drugs on judgement bias at lower doses, may be attributed to side effects or to the complex nature of adrenergic drug effects. The effect of depressant and anxiogenic drugs state was greater than the effect of antidepressant and anxiolytic drugs, and therefore larger sample sizes may be required when testing the efficacy of potential pharmacological treatments for mood disorders. However, if consideration is given to these potential shortcomings, the judgement bias task for which there is evidence of construct validity and now of predictive validity appears to be a viable measure of whether the neurobiological state of non-human animals is indicative of a positive or negative affective state.
