# Supplementary material for: Pharmacological manipulations of judgement bias: A systematic review and meta-analysis
Source: Neurosci Biobehav Rev. 2020 Jan;108:269–86. doi: 10.1016/j.neubiorev.2019.11.008 (PMC6966323; doi:10.1016/j.neubiorev.2019.11.008)
Supplement: Supplementary file 1 [file mmc1.docx]

**Supplementary Material**

Details of the literature searches:

# ***Main Database Searches***

The following search was conducted using Scopus (on 12^th^ July 2019):

( TITLE-ABS-KEY ( ( "Cognitive bias*" OR "judgement bias*" OR "judgement bias*" OR "Cognitive affective bias*" ) AND ( "pessimis*" OR "optimis*" OR "valence" OR "mood*" OR "emotion*" OR "affective state*" OR "emotional state*" OR "ambig*" ) AND ( "animal*" OR "animal welfare" ) ) AND ( PUBYEAR > 2016 ) ) OR ( TITLE-ABS-KEY ( ( "Cognitive bias*" OR "judgement bias*" OR "judgement bias*" OR "Cognitive affective bias*" ) AND drug* ) )

The following search was conducted using Web of Science (on 12^th^ July 2019):

(TS=( ( "Cognitive bias*" OR "judgement bias*" OR "judgement bias*" OR "Cognitive affective bias*" ) AND ( "pessimis*" OR "optimis*" OR "valence" OR "mood*" OR "emotion*" OR "affective state*" OR "emotional state*" OR "ambig*" ) AND ( "animal*" OR "animal welfare" ) ) AND ( PY=(2017-2019) ) ) OR ( TS=( ( "Cognitive bias*" OR "judgement bias*" OR "judgement bias*" OR "Cognitive affective bias*" ) AND drug* ) )

Indexes: SCI-EXPANDED, SSCI, A&HCI, CPCI-S, CPCI-SSH, BKCI-S, BKCI-SSH, ESCI, CCR-EXPANDED, IC.

The following search was conducted within the titles, abstracts, and keywords of research articles using Scopus and Web of Science (on 2^nd^ November 2016 and 13^th^ November 2017):

("Cognitive bias*" OR "judgment bias*" OR "judgement bias*" OR "Cognitive affective bias*")AND("pessimis*" OR "optimis*" OR "valence" OR ”mood*" OR "emotion*" OR "affective state*" OR "emotional state*" OR "ambig*") AND ("animal*" OR "animal welfare")

# ***Subject database searches***

The following databases were searched via the University of New South Wales (12-14 July 2019):

All APA Psychology databases via the Ovid platform including PsycINFO, PsycARTICLES, PsycBOOKS, PsycEXTRA and PsycTESTS using the following search:

(("Cognitive bias*" or "judgement bias*" or "judgement bias*" or "Cognitive affective bias*") and ("pessimis*" or "optimis*" or "valence" or "mood*" or "emotion*" or "affective state*" or "emotional state*" or "ambig*") and ("animal*" or "animal welfare")).mp. [mp=ti, ab, td, hw, tc, id, ot, tm, mh, tx, ct]

The EMBASE database using the following search:

(("Cognitive bias*" or "judgement bias*" or "judgement bias*" or "Cognitive affective bias*") and ("pessimis*" or "optimis*" or "valence" or "mood*" or "emotion*" or "affective state*" or "emotional state*" or "ambig*") and ("animal*" or "animal welfare")).mp. [mp=title, abstract, heading word, drug trade name, original title, device manufacturer, drug manufacturer, device trade name, keyword, floating subheading word, candidate term word]

The Medline database using the following search:

(("Cognitive bias*" or "judgement bias*" or "judgement bias*" or "Cognitive affective bias*") and ("pessimis*" or "optimis*" or "valence" or "mood*" or "emotion*" or "affective state*" or "emotional state*" or "ambig*") and ("animal*" or "animal welfare")).mp. [mp=title, abstract, original title, name of substance word, subject heading word, floating sub-heading word, keyword heading word, organism supplementary concept word, protocol supplementary concept word, rare disease supplementary concept word, unique identifier, synonyms]

# ***Grey literature***

The following grey literature databases searches were conducted between (14-16 July 2019), as follows:

The ProQuest Dissertation and Thesis database using the following searches:

noft(affect*) AND noft(bias*) AND noft(pharmacol*)

noft(affective) AND noft(bias*) AND noft(animal*)

Google Dataset Search using the following keyword string:

( "Cognitive bias*" OR "judgement bias*" OR "judgement bias*" OR "Cognitive affective bias*" )

The Dimensions database using the following search:

affect* AND bias AND pharmacol* AND (animal OR welfare*)

# ***Snowballing***

The following review articles were used for snowballing in Scopus (on 17^th^ July 2019):

1. Clegg, I. (2018). Cognitive bias in zoo animals: An optimistic outlook for welfare assessment. Animals, 8(7), 104.
2. Crump, A., Arnott, G., and Bethell, E. (2018). Affect-driven attention biases as animal welfare indicators: review and methods. Animals, 8(8), 136.
3. Hales, C. A., Stuart, S. A., Anderson, M. H., and Robinson, E. S. (2014). Modelling cognitive affective biases in major depressive disorder using rodents. British journal of pharmacology, 171(20), 4524-4538.
4. Marino, L. (2017). Thinking chickens: a review of cognition, emotion, and behavior in the domestic chicken. Animal Cognition, 20(2), 127-147.

# ***Article Screening***

Decision tree used to screen articles identified through the literature search:
